# Gut Microbiota: Critical Controller and Intervention Target in Brain Aging and Cognitive Impairment

**DOI:** 10.3389/fnagi.2021.671142

**Published:** 2021-06-25

**Authors:** Hui Li, Junjun Ni, Hong Qing

**Affiliations:** Key Laboratory of Molecular Medicine and Biotherapy, School of Life Sciences, Beijing Institute of Technology, Beijing, China

**Keywords:** gut microbiota, brain aging, machine learning, cognitive impairment, Alzheimer’s disease, diet, probiotics

## Abstract

The current trend for the rapid growth of the global aging population poses substantial challenges for society. The human aging process has been demonstrated to be closely associated with changes in gut microbiota composition, diversity, and functional features. During the first 2 years of life, the gut microbiota undergoes dramatic changes in composition and metabolic functions as it colonizes and develops in the body. Although the gut microbiota is nearly established by the age of three, it continues to mature until adulthood, when it comprises more stable and diverse microbial species. Meanwhile, as the physiological functions of the human body deteriorated with age, which may be a result of immunosenescence and “inflammaging,” the guts of elderly people are generally characterized by an enrichment of pro-inflammatory microbes and a reduced abundance of beneficial species. The gut microbiota affects the development of the brain through a bidirectional communication system, called the brain-gut-microbiota (BGM) axis, and dysregulation of this communication is pivotal in aging-related cognitive impairment. Microbiota-targeted dietary interventions and the intake of probiotics/prebiotics can increase the abundance of beneficial species, boost host immunity, and prevent gut-related diseases. This review summarizes the age-related changes in the human gut microbiota based on recent research developments. Understanding these changes will likely facilitate the design of novel therapeutic strategies to achieve healthy aging.

## Introduction

The average human lifespan expectancy in most countries is longer than ever before, benefiting from the achievements of modern medicine and lifestyle improvements. However, this has led to the rapid growth of the world’s aging population (60 and older) (Riaz Rajoka et al., 2018). Aging is usually associated with various high-risk and long-lasting diseases, such as cancer, neurodegenerative disorders, diabetes, and metabolic syndrome, that seriously affect the life quality of the elderly (Dillin et al., 2014). The question of how to increase life expectancy while also reducing the duration and severity of morbidity poses a serious challenge for society.

Over 100 years ago, Elie Metchnikoff linked the gut microbiota with human health and aging for the first time, theorizing that health and lifespan could be enhanced and prolonged by regulating the gut microbiome *via* the consumption of host-friendly bacteria found in yogurt (Mackowiak, 2013). The gut microbial community, a “super organism” residing in the human intestinal tract, comprises 1 × 10^13–14^ microorganisms, including approximately 500-1,000 bacterial species, that form a mutually beneficial symbiosis with their human hosts (Qin et al., 2010; Biagi et al., 2012). Besides aiding in digestive processes and food absorption, other functions of this super organism include synthesizing essential amino acids and vitamins, metabolizing fibers into short-chain fatty acids (SCFAs), maintaining the integrity of the intestinal mucosal barrier, regulating host immunity, and protecting the host from pathogen attack (Gill et al., 2006), all of which contribute to the maintenance of human health and well-being. Contrasting with this, gut dysbiosis can be a major contributor to physiological deterioration and the onset of geriatric diseases in humans (Kim and Jazwinski, 2018).

The application of 16s rRNA sequencing, based on second-generation, high-throughput technology, has greatly advanced the study of gut microbiota and opened the door for modern research into intestinal microecology (Dave et al., 2012; Bischoff, 2016). Additionally, the development of metagenomics has made it possible to define the gene content and encoded functional attributes of the gut microbiome in humans (Gill et al., 2006). Owing to the large amount of sequencing data generated in gut microbiota research, how to acquire useful information from the massive amounts of data has become an urgent task. The rise of machine learning provides new ideas for the study of gut microbiota. Various machine learning approaches, which can effectively identify taxonomic or functional signatures, have recently been applied to reveal the aging-related changes in the microbial community and identify the bacterial genera associated with these changes (Hopkins et al., 2001; Odamaki et al., 2016; Bian et al., 2017).

In this review, we summarize recent research findings on the role of gut microbiota in the aging process, and the application of machine learning for the analysis of age-related changes in gut microbe composition. We also highlight the link between bacterial communities and human health and the mechanisms underlying how the gut microbiota influence the development of age-related diseases, particularly cognitive impairment disorders, as well as the contribution of diet and probiotics/prebiotics to the maintenance of a beneficial gut microbiome to achieve healthy aging.

## Age-Related Changes in the Human Gut Microbiota

**FIGURE 1:**
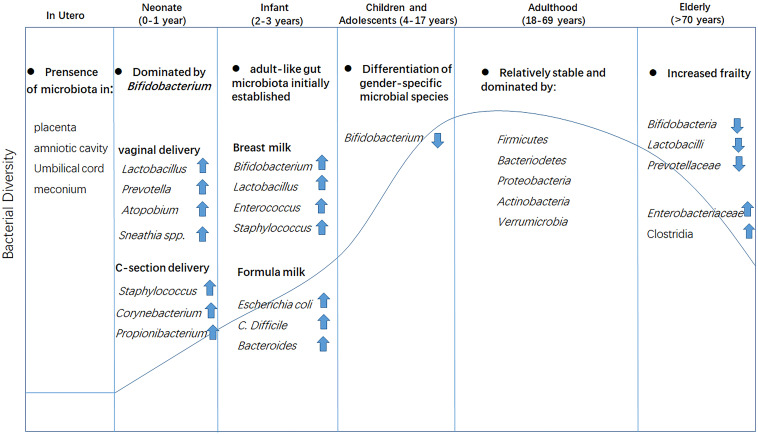
Changes in gut microbiota composition at different life stages. Microbiota colonization may begin in the uterus as evidenced by the presence of bacteria in the placenta, amniotic cavity, umbilical cord, and meconium. The mode of delivery at birth has a direct effect on the bacterial community. The gut microbiota is initially established at approximately 3 years of age and matures at the onset of adulthood with the establishment of dietary habits, leading to a more diverse and stable gut microbial community. In old age, the gut microbiota is drastically altered and diversity is greatly reduced.

The presence of bacteria in the placenta, amniotic cavity, umbilical cord, and meconium suggests that the relationship between humans and microbes may begin in the uterus (Jimenez et al., 2005; Collado et al., 2016). The mode of delivery at birth directly influences the diversity of the microbiota in an infant (Rutayisire et al., 2016). Vaginally delivered infants have a more diverse and healthier bacterial community in their gut than those born by cesarean section (Jakobsson et al., 2014). In the first two to 3 years after birth, the gut microbiota of infants undergoes marked changes, influenced mainly by their feeding pattern (formula milk, breast milk, or solid food) (Cong et al., 2016; Kundu et al., 2017). Subsequently, the composition of the bacterial community remains relatively stable until adulthood, when it becomes fully established (Lynch et al., 2015). Generally, the most abundant phyla in a healthy adult gut are *Firmicutes*, *Bacteroidetes*, *Proteobacteria*, *Actinobacteria*, and *Verrucomicrobia*, with *Firmicutes* and *Bacteriodetes* accounting for almost 80% of this abundance (Eckburg et al., 2005). In adults, diet becomes the main influencing factor, i.e., the function of the gut microbiome changes from the lactate utilization seen in the infant to plant polysaccharide breakdown, xenobiotic degradation, and vitamin biosynthesis (Riaz Rajoka et al., 2018). With the deterioration of physiological functions (such as reduced intestinal functionality, chewing problems, and reduced immunity) with aging, the composition of the gut microbiota in the elderly changes markedly. Compared with healthy younger adults, the microbiota in the elderly gut is generally characterized by a reduced abundance of some beneficial genera, such as *Bifidobacterium* and *Lactobacillus*, and a marked increase in that of pro-inflammatory commensal microbes, such as Enterobacteriaceae and Clostridia (O’Toole and Jeffery, 2015; Odamaki et al., 2016; Figure 1). Aging is often accompanied by a decline in the diversity of the gut microbiota (Bischoff, 2016), which may be linked with the increased frailty index of the elderly (van Tongeren et al., 2005; Jackson et al., 2016). Cognitive impairment is a common disorder and is usually accompanied by changed microbiota in the elderly compared with that of a healthy elderly person. Clinical evidence has connected the gut microbiota dysbiosis with cognitive deficit in the elderly, because subjects with cognitive decline and brain amyloidosis had lower abundance of anti-inflammatory *E. rectale* and higher abundance of pro-inflammatory *Escherichia/Shigella* in their stools when compared with that of control subjects (Cattaneo et al., 2017). It was proved that the abundance of *Lactobacillales* members was positively while *Enterobacteriaceae* and *Porphyromonadaceae* were negatively associated with cognition in the elderly (Bajaj et al., 2016). This evidence have disclosed a direct link between microbes and cognitive conditions in the elderly.

Although the composition of the gut microbiome of longevous elderly individuals (mainly centenarians) also changes, its diversity and beneficial species are retained (Ragonnaud and Biragyn, 2021), partially explaining how they maintain homeostasis and health, and, consequently, achieve their longevity. A recent study of centenarians in Italy revealed a cumulative decline in the abundance of a core microbiota with aging, but the age-related enrichment of subdominant taxa, including *Ruminococcaceae*, *Lachnospiraceae*, and *Bacteroidaceae* families, was boosted in longevity (Biagi et al., 2016). Similar studies in China have suggested that the gut microbiota of long-lived individuals (>90 years old) displays greater robustness and abundance than that of younger individuals (65-70 years old) as indicated by greater alpha-diversity (Kong et al., 2016; Wang N. et al., 2019). Eleven of the top 50 features were shared between these two independent studies, including community richness and significant changes in members of *Blautia*, *Clostridium* cluster XIVa, *Faecalibacterium*, *Escherichia*, and *Shigella*, and unclassified Lachnospiraceae, suggestive of a similar characteristic in the microbiota composition shared by longevous people from different regions. However, in most cases, the microbiota of humans display great inter-individual variations, as ethnicity, geography, and lifestyle also influence age-related changes in gut microbe composition (Ragonnaud and Biragyn, 2021). The typical diet in the United States is rich in protein, whereas in Malawi and among Amerindian populations the diet is dominated by corn and cassava. Accordingly, the microbes in the guts of inhabitants of metropolitan areas in the United States were significantly different from those of residents of rural Malawian communities and Amerindians from the Amazonas of Venezuela (Yatsunenko et al., 2012). Japanese adults (21–69 years old) have a greater abundance of *Blautia* and *Bifidobacterium* and a relatively lower abundance of *Bacteroidetes*-related genera compared with adults of other nations (Odamaki et al., 2016), including the United States, Colombia, South Korea, and China (Nam et al., 2011; Escobar et al., 2014). The abundance of *Clostridium* cluster XIVa was lower in Japanese, Italian, and Finnish elderly (Hayashi et al., 2003; Mueller et al., 2006; Makivuokko et al., 2010), but was higher in elderly Germans (Mueller et al., 2006). Meanwhile, the abundance of *Bacteroides* was increased in the Austrian elderly (Zwielehner et al., 2009), whereas in the Italian elderly an inverse trend was noted (Mueller et al., 2006).

To identify the sequential changes occurring in the gut microbiota during aging, a large number of studies have recently employed machine learning methods. Machine learning has unique advantages, including in cluster analysis of microbiota (Zhang et al., 2017), the identification and classification of microbiota (Wang et al., 2007; Zielinski et al., 2017; Oudah and Henschel, 2018), and the prediction of host phenotype (Johnson et al., 2016; Thompson et al., 2019). Using permutational multivariate analysis of variance (PERMANOVA), a method used in the supervised machine learning method, Odamaki et al. (2016) investigated fecal samples obtained from 367 healthy Japanese individuals aged less than 104 years, and revealed that the gut microbiota of those aged less than 20 years matured with age, while that of subjects over 70 years of age changed into the elderly type. The authors found that the aging process was accompanied by an increased abundance of *Bacteroides*, *Eubacterium*, and *Clostridiaceae*. Sequential changes occurred in the relative abundance of *Bacteroides*, Lachnospiraceae, and Bifidobacteria in the gut microbiota during childhood and adolescence (<20 years old), while *Megamonas* and *Peptoniphilus* were relatively enriched in the elderly (>70 years old). Using the same data samples, Xu et al. (2019) applied an unsupervised algorithm called Sample Progression Discovery (SPD) on genera abundance profile, and identified 35 genera associated with a continuous progression in the composition of the human gut microbiota with aging. Among these 35 genera, the abundance of some beneficial genera taxa, such as *Lactobacillus*, *Oscillospira*, *Oxalobacter*, *Prevotellaceae*, *Parascardovia*, and *Butyrivibrio*, increased with age, but decreased in the extremely elderly. In contrast, some taxa that are frequently linked with inflammation and diseases, including *Parvimonas*, *Anaerotruncus*, *Corynebacterium*, *Lachnospiraceae*, *Desulfovibrio*, *Bilophila wadsworthia*, *Odoribacter*, and *Butyricimonas*, showed monotonically increasing patterns with respect to aging. Yatsunenko et al. (2012) investigated 531 fecal samples of individuals from three different countries using Spearman’s rank correlation and Random Forest methods and reported a decline in the abundance of *Bifidobacterium longum* species but an increase in the overall bacterial diversity with aging (0–70 years). However, another study obtained different results from the gut microbiota of more than 1,000 healthy Chinese individuals aged from 3 to over 100 years using PERMANOVA. These authors reported that the gut microbiota differed little among individuals from the ages of 30 to >100 and that the major between-group differences in gut microbiota profiles were found before 20 years of age (Bian et al., 2017). The similarity between the microbial diversity of the elderly and that of people decades younger may have been due to the participants being mostly very healthy or small sample size.

Collectively, despite the considerable inter-individual variability and the impact of external factors such as the dietary habits and geography/culture of the host (Claesson et al., 2012; Yatsunenko et al., 2012), it is increasingly clear that an intrinsic aging progression in the composition of human gut microbiota exists (Xu et al., 2019; Ragonnaud and Biragyn, 2021; Figure 1). In contrast, less is known of the mechanisms underlying these changes owing to the complicated physiological conditions and huge differences between individuals.

## The Role of the Microbiota During Aging

The crucial influence of microbial composition on the rate and quality of aging is well-documented (Candela et al., 2014; Saraswati and Sitaraman, 2014). The changes in gut microbiome composition occur gradually (O’Toole and Jeffery, 2015). The fecal microbiota differs significantly between community-dwelling individuals and those living in long-term care nursing facilities (van Tongeren et al., 2005; Claesson et al., 2012; Kinross and Nicholson, 2012; Collino et al., 2013), consistent with its role in healthy aging.

Immunosenescence, which refers to the gradual deterioration of the immune system with age, represents an important feature of the aging process. The chronic and low-grade activation of the innate and adaptive immune systems, also known as “inflammaging,” is linked to immunosenescence, as evidenced by the persistence of NF-κB-mediated inflammation and the reduction of naïve CD4^+^ T cell numbers (Franceschi, 2007; Claesson et al., 2012). Although the cause of inflammaging remains poorly understood, emerging evidence has connected it with reduced autoimmune tolerance of the gut microbiota and changes in its composition with aging (Guigoz et al., 2008). The gut microbiota is pivotal for maintaining the immune homeostasis of the human intestine (Honda and Littman, 2016). This can be directly evidenced in the germ-free (GF) mice, where the absence of gut microbes leads to the impaired the development and maturation of the immune system, while their presence induces the production of interleukin 10 (IL-10) and transforming growth factor beta (TGF-β)-producing regulatory T cells (Tregs), T helper 17 (Th17) cells, type-2 lymphoid innate cells (ILC2), and immunoglobulin A (IgA)-secreting B cells (Hansson et al., 2011; Atarashi et al., 2013, 2015; Furusawa et al., 2013; Satoh-Takayama et al., 2020). Besides, some beneficial bacterial species and their metabolites, particularly SCFAs and their precursors, have been reported to decline during aging in humans, and may be a primary cause of frailty in the elderly (Ragonnaud and Biragyn, 2021). Certain constituents of the gut microbiota were demonstrated to be predictors of exceptional human longevity (Vaiserman et al., 2017). The longevity of both Chinese and Italian centenarians has been positively associated with an abundance of beneficial commensals, such as *Clostridium* cluster XIVa, *Akkermansia muciniphila*, *Christensenellaceae*, and *Bifidobacteria* (Biagi et al., 2016; Kong et al., 2016). *Clostridium* cluster XIVa includes many genera involved in SCFAs production (van den Abbeele et al., 2013), while *A. muciniphila* helps maintain intestinal epithelial integrity by inducing the mucin production, supports beneficial SCFA-producing bacteria, and aids in reducing inflammation and metabolic impairments, such as insulin resistance (Schneeberger et al., 2015; Bodogai et al., 2018). The abundance of *Christensenellaceae* in the human gut shows an inverse correlation with the body mass index (BMI) and can help mitigate the effects of some inflammatory diseases in humans (Waters and Ley, 2019). *Bifidobacterium* can modulate the PH of the body and facilitate the digestion and absorption of essential nutrients and synthesis of vitamins through producing lactic acid, pyruvic acid, and butyrate; besides, it can inhibit the growth of pathogenic bacteria and enhance the immune response of the host (Yusof et al., 2000). These beneficial bacteria play important roles in gut homeostasis, and may help sustain a healthy state during aging.

Overall, the reduction of beneficial bacteria and enrichment of pro-inflammatory species, along with reduced gut microbial diversity, can exert detrimental effects on healthy aging and, consequently, longevity.

## The Brain-Gut-Microbiota Axis

The gut and brain are closely connected through a complex bidirectional communication system, known as the brain-gut-microbiota (BGM) axis, which primarily includes neural, immune, metabolic, and endocrine pathways (Martin et al., 2018).

The brain can directly or indirectly affect the composition and function of the gut microbiota by releasing signal molecules through lamina propria cells (intestinal chromaffin cells, neurons and immune cells) or modulating gastrointestinal motility, secretions, and intestinal permeability (Cryan et al., 2019). Inversely, the gut microbiota can influence the function, behavior, and health of the brain, and is suggested to be a key regulator of brain development, aging, and neurodegeneration (Dinan and Cryan, 2017). A normal gut microbiota–brain interaction is essential for the maintenance of a healthy physiological condition and normal cognitive functions.

Gut microbes can communicate with the brain *via* neuronal signaling that involves the central nervous system (CNS), autonomic nervous system, and enteric nervous system (ENS), often involving the stimulation of the vagus nerve. The vagus nerve is a bundle of parasympathetic motor and sensory fibers that provide a direct means of neurocommunication between the ENS and the CNS (Forsythe et al., 2014; Fung et al., 2017). Studies have found the genes encoding γ-aminobutyric acid (GABA) receptors are highly expressed in vagal afferent neurons and the nodose ganglion in the gastrointestinal tract of mice. The vagus nerve can sense GABA signals in the intestine and transmit them to the brain, as evidenced by the increased numbers of GABA_*A*_ receptors and glutamic acid decarboxylase (GAD)-positive cells in the cerebral cortex following stimulation of the peripheral end of the vagus nerve (Marrosu et al., 2003; Neese et al., 2007; Egerod et al., 2018). Moreover, supplementation of the probiotic *Lactobacillus rhamnosus* JB-1 to mice can increase the mRNA level of the GABA_*B*_ receptor B1 subunit in the cerebral cortex and that of the GABA_*A*_ receptor α2 subunit in the prefrontal cortex and apricot kernel; suppress the stress-induced increase in corticosterone levels and alleviate anxiety and depression in mice; however, these beneficial effects were abolished when the mice were vagotomized (Bravo et al., 2011).

The effects of microbiota on CNS functions can also be mediated through the circulatory system *via* several microbial-derived molecules, including neurotransmitters, hormones, their precursors, and SCFAs. Some metabolites can pass through the intestinal barrier and enter the systemic circulation, and some can even cross the blood–brain barrier (BBB), thereby regulating neurological functions (O’Mahony et al., 2015; Koh et al., 2016; Sharon et al., 2016). The BBB forms during gestation and controls the passage and exchange of molecules and nutrients between the circulatory system and the brain parenchyma, which ensures CNS homeostasis (Braniste et al., 2014). The gut microbiota is important for the maintenance of BBB integrity, as evidenced by the increased BBB permeability and lower expression of the tight junction proteins occludin and claudin-5 in different brain regions of GF mice, while exposure of GF adult mice to a pathogen-free gut microbiota decreased BBB permeability and up-regulated the expression of tight junction proteins (Braniste et al., 2014). SCFAs are biologically active molecules that are mainly produced by beneficial intestinal microbes through the digestion of dietary fiber. These molecules can transverse the BBB and serve as key signaling metabolites in BBB development and maintenance via entering cells and acting as histone deacetylase inhibitors for epigenetic modulation or by binding to G protein-coupled receptor (GPR) 41 and/or GPR43 receptors on the cell membrane (Braniste et al., 2014; Michel and Prat, 2016). Although some gut neurotransmitters, such as serotonin (5-hydroxytryptamine, 5-HT), GABA, and dopamine, cannot cross the BBB, they can act on the vagus nerve or affect signaling in the peripheral nervous system, thereby eventually influencing brain functions (Wu et al., 2020). Serotonin, a well-characterized neurotransmitter known for its roles in modulating neural activity and a wide range of neuropsychological processes, is also an important regulator of gastrointestinal motility and cardiovascular function, among other functions (Berger et al., 2009). However, more than 90% of the body’s 5-HT is synthesized by the enterochromaffin cells (ECCs) of the gastrointestinal tract. Metabolites such as SCFAs and 2Bas that are produced by indigenous spore-forming microbes have been demonstrated to promote the biosynthesis and release of 5-HT by ECCs (Yano et al., 2015). Tryptophan, the 5-HT precursor, is an essential amino acid that must be supplied in the diet. The gut microbiota contributes to the peripheral availability of tryptophan. Once absorbed from the gut, it enters the circulatory system, crosses the BBB *via* large amino acid transporters, and is converted to 5-HT in the CNS.

Neuroimmune signaling is also an important pathway for the communication between the gut microbiota and the CNS. In general, microbial-derived metabolites or other components mediate immune system activities. SCFAs can promote microglial maturation and are needed for the maintenance of mature microglia (Erny et al., 2015). Microglia are the resident macrophages and major immune defense cells of the CNS. GF mice have fewer microglia, and their morphology and function are abnormal compared with those of specific-pathogen-free (SPF) mice; however, these effects can be reversed by the administration of SCFAs to GF mice, and this may be primarily dependent on the activation of GPR43 by SCFAs (Erny et al., 2015). In addition, microbiota-derived microbial-associated molecular patterns (MAMPs) derived from microbiota, such as lipopolysaccharide (LPS), bacterial lipoprotein (BLP), flagellin, and cytosine-phosphate-guanosine (CpG) DNA, can activate the immune cells of the peripheral innate immune system and subsequently induce the release of numerous pro-inflammatory cytokines, such as interleukin-1-β (IL-1β), interleukin-6 (IL-6), and tumor necrosis factor-α (TNF-α) (Sampson and Mazmanian, 2015). These pro-inflammatory cytokines can act on receptors in afferent nerves or cross the BBB and enter the brain parenchyma, eventually leading to changes in neurological functions in the CNS (Dantzer et al., 2000; Fung et al., 2017).

Recently, a number of studies have highlighted the potential role of bacteriophages as regulators of aging and neurodegeneration. Bacteriophages are the most abundant members of the microbial community, and have the potential to shape gut bacterial communities and modulate microbiota stability (Gogokhia et al., 2019). Bacteriophages can influence the development of human diseases either directly through interactions with eukaryotic cells and proteins, or indirectly by inducing changes in the abundance of certain bacteria and increasing the levels of pathogen-associated molecular patterns (PAMPs). Many bacteriophages have the ability to maintain intestinal barrier integrity by embedding themselves within the mucus layer and controlling invasive bacterial populations (Barr et al., 2013), and can also alter mucosal immunity, thereby influencing mammalian health (Gogokhia et al., 2019). In a rodent model, bacteriophage administration induced shifts in the gut microbiota, leading to increased intestinal permeability and triggering chronic inflammation (Tetz and Tetz, 2016). Recent studies have demonstrated that bacteriophages are present in the cerebrospinal fluid (CSF) of subjects with neurodegenerative pathologies (Tetz and Tetz, 2018), and that PD patients display a greater abundance of lytic *Lactococcus* phages and a 10-fold reduction in neurotransmitter-producing *Lactococcus* bacteria compared with that in healthy individuals, suggestive of an association with and the possible role of phages in neurodegeneration (Tetz et al., 2018). Bacteriophages may affect the CNS through pathways involved in increasing intestinal permeability, altering the abundance of bacteria that are important for the maintenance of a healthy CNS, and inducing a chronic systemic inflammatory response (Tetz and Tetz, 2018). However, substantial research is still needed to elucidate these underlying mechanisms.

## Gut Microbiota and Cognitive Impairment

Unimpaired cognitive skills are crucial for the daily functioning of older people. However, some of these cognitive skills including memory, learning and problem-solving activities, decline during aging. Multiple risk factors have been proposed to be related to cognitive impairment (Klimova et al., 2017). Studies have increasingly emphasized the influence of microorganisms on host behavior and cognitive function, such as those involving GF animal models in which behavioral disorders and reduced cognitive function are prominent (Lee et al., 2020).

**FIGURE 2:**
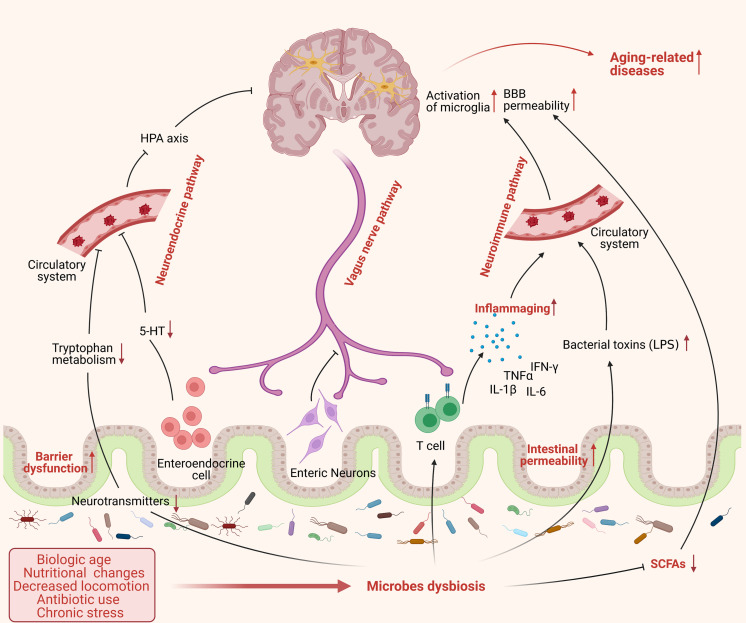
The dysbiosis of gut microbes in aging process. Aging is accompanied by significant changes in lifestyle, such as biological age, nutritional changes, decreased locomotion, antibiotic use, and chronic stress, which may cause an alteration of microbes’ composition and exacerbate the dysbiosis of gut microbiota in frail elderly people. Microbes dysbiosis in aging shows a severe decrease of beneficial bacteria, such as SCFA-producing bacteria, and increased pathogenic bacteria, such as Enterobacteriaceae, affecting gut physiology and leading to reduced intestinal motility, gut-barrier dysfunction, and increased intestinal permeability. Furthermore, the dysbiosis of microbiota could influence the brain function through various routes including: (1) promote the release of pro-inflammatory cytokines and bacterial toxins to the circulatory system, such as LPS, causing systemic inflammation via neuroimmune pathway; (2) inhibit the transmission of neural signal from enteric nervous system (ENS) to central nervous system (CNS) mainly via vagus nerve; (3) suppress the production and release of microbial neurotransmitters and hormones to circulatory system and subsequently to brain through neuroendocrine pathway. All these factors cause a chain of detrimental events to the brain that enhancing the risk of developing aging-related pathologies. (This figure was created with BioRender.com).

Gut microbiota can play an intrinsic role in aging-related impairments in a range of cognitive processes. The dysbiosis of gut microbiota is involved in aging-related diseases in multiple pathways (Figure 2). Aging is associated with low-grade inflammation, and prolonged exposure of the brain to inflammatory cytokines can impair cognition. Studies on aged mice have shown that acute inflammation can induce memory defects (Chen et al., 2008; Barrientos et al., 2009). Microbial dysbiosis or increased gut permeability with aging has similarly been linked with inflammation through inducing the production and release of bacterial components; such as LPS, lipoproteins, and double-stranded RNA into the bloodstream. This leads to the activation of the immune system and the release of pro-inflammatory cytokines such as TNF-α, IL-1β, and IL-6 (Sampson and Mazmanian, 2015; Komanduri et al., 2019). Age-associated cognitive decline may also be attributed to a decrease in synaptic connections in the brain (Vanguilder et al., 2010). Meanwhile, gut microbes are capable of producing neurotransmitters (such as GABA, acetylcholine, and dopamine) and neurotrophic factors (such as brain-derived neurotrophic factor [BDNF] and nerve growth factor [NGF]), which are essential for the transmission of nerve signals (Diaz Heijtz et al., 2011; Sampson and Mazmanian, 2015). *Lactobacilli* and *Bifidobacteria* can alleviate anxiety and depression-like symptoms by converting glutamate in the gut into GABA (Yunes et al., 2016). The numbers of *Alcaliganeceae* and *Porphyromonadaceae* were shown to be positively correlated with cognitive decline (Caracciolo et al., 2014). Besides, gut microbiota can also affect cognition through promoting oxidative stress, as evidenced by the sharp rise in the abundance of cyanobacteria in Alzheimer’s disease (AD) patients. The cyanobacterial-derived metabolite (β-N-methylamino-L-alanine) can activate glutamate receptors, leading to oxidative stress in neurons and, ultimately, neuronal apoptosis (Banack et al., 2010).

Many preclinical studies have highlighted the crucial role of alterations in brain-gut-microbiome communication not only in the pathogenesis and pathophysiology of classic brain-gut disorders such as irritable bowel syndrome (IBS), and obesity, but also in psychiatric and neurological disorders, such as depression, anxiety neurosis, AD, and Parkinson’s disease (PD) (Collins et al., 2012; Martin et al., 2018; Table 1).

**TABLE 1:** Alterations of the gut microbiota in different geriatric diseases.

Disease	Subjects	Gut microbiota alterations	References
Alzheimer’s disease	AD human patients	*Bacteroides*↓; *Ruminococcus*↑; *Actinobacteria*↑; *Lachnospiraceae*↓	Zhuang et al., 2018
	AD human patients	*Escherichia/Shigella* (pro-inflammatory taxon) ↑; *E. rectale* (anti-inflammatory taxon) ↓	Cattaneo et al., 2017
	AD human patients	*Butyrivibrio*/*Eubacterium*/*Clostridium* (key butyrate-producing species)↓; *O. splanchnicus*↑; *K. pneumonia*↑	Haran et al., 2019
	AD human patients	microbial richness and diversity↓; *Firmicutes*↓; *Bacteroidetes*↑; *Bifidobacterium*↓	Vogt et al., 2017
	APP/PS1 mice	Microbiota diversity↓; *Helicobacteraceae*↑; *Desulfovibrionaceae*↑; *Odoribacter*↑; *Helicobacter*↑; *Prevotella*↓	Shen et al., 2017
	5 × FAD mice	*Firmicutes:Bacteroidetes* ratio↑; *Clostridium leptum*↑	Brandscheid et al., 2017
Parkinson’s disease	PD human patients	Prevotellaceae↓; Enterobacteriaceae↑	Scheperjans et al., 2015
	PD human patients	Intestinal permeability↑; *E. coli* bacteria↑;	Forsyth et al., 2011
	PD human patients	*Blautia*↓; *Coprococcus*↓; *Roseburia*↓; *Faecalibacterium*↓; *Ralstonia*↑	Keshavarzian et al., 2015
	PD human patients	*Lactobacillus*↑; *Faecalibacterium*↓; *Coprococcus*↓; *Blautia*↓; *Bifidobacteriu*↑;*Verrucomicrobiaceae*↑	Gerhardt and Mohajeri, 2018
	PD human patients	*Bacteroides*↑; *Prevotella*↓; *Parabacteroides*↑; *Verrucomicrobia*↑; *Akkermansia*↑; *Butyricimonas*↑; *Veillonella*↑; *Odoribacter*↑; *Mucispirillum*↑; *Bilophila*↑; *Enterococcus*↑; *Lactobacillus*↑	Lin et al., 2019
Hypertension	Hypertension patients	Microbial richness and diversity↓; *Prevotella*↑; *Klebsiella*↑; *Porphyromonas*↑; *Actinomyces*↑; *Faecalibacterium*↓; *Oscillibacter*↓; *Roseburia*↓; *Bifidobacterium*↓; *Coprococcus*↓; *Butyrivibrio*↓	Li et al., 2017
	Hypertension patients and SHRs rat	Microbial richness and diversity↓; *Firmicutes*/*Bacteroidetes* ratio↑; Acetate- and butyrate-producing bacteria (*Coprococcus* and *Pseudobutyrivibrio*)↓; lactate-producing bacteria (*Streptococcus* and *Turicibacter*)↑	Yang et al., 2015
Atherosclerosis	Atherosclerosis patients	*Collinsella*↑; *Eubacterium*↓; *Roseburia*↓; *Bacteroides*↓	Karlsson et al., 2012
	Atherosclerosis patients	*Escherichia coli*↑; *Klebsiella* spp.↑; *Enterobacter aerogenes*↑; *Streptococcus* spp.↑;*Lactobacillus salivarius*↑; *Solobacterium moorei*↑; *Atopobium parvulum*↑; *Bacteroides* spp.↓; *Prevotella copri*↓; *Alistipes shahii*↓	Jie et al., 2017
Type 2 diabetes mellitus	T2DM patients	*Firmicutes*/*Bacteroidetes* ratio↓; *Clostridia*↓; *Proteobacteria*↑; *Bacilli*↑	Larsen et al., 2010
	T2DM patients	Verrucomicrobiae↓; butyrate producing bacteria (*Akkermansia muciniphila* and *Faecalibacterium prausnitzii*)↓	Zhang et al., 2013
	T2DM patients	*Lactobacillus*↑; *Bifidobacteria*↓	Sedighi et al., 2017
		*Faecalibacterium prausnitzii*↓; *Akkermansia muciniphila*↓	Fassatoui et al., 2019

### Alzheimer’s Disease

Alzheimer’s disease is a progressive neurodegenerative syndrome and the most common cause of dementia. The pathogenesis of AD includes the excessive aggregation of amyloid-β (Aβ) peptides, the presence of neurofibrillary tangles (NFTs) induced by the hyperphosphorylation of tau protein, neuroinflammation, and metabolic disturbances; however, the mechanisms underlying the pathogenesis of AD is not fully understood (Jagust, 2018).

Accumulating evidence supports a close connection between gut microbiota dysbiosis and AD (Jiang et al., 2017). For example, the structure of the mucosa of the small intestine of AD transgenic mice differs from that of normal mice, while the number of Gram-negative bacteria in the colon is also significantly reduced in the AD mice (Karri et al., 2010). The McCarthy survey showed that the gut microbiota was altered in 85% of dementia patients in the United Kingdom (Xiao et al., 2014). Recent studies revealed that fecal samples of AD patients display higher relative abundances of taxa known to promote a pro-inflammatory state, such as *Escherichia Shigella*, *Odoribacter splanchnicus*, and *Klebsiella pneumonia*, and reductions in key butyrate-producing anti-inflammatory species, such as members of the genera *Butyrivibrio* and *Eubacterium*, compared with those of non-AD individuals (Cattaneo et al., 2017; Haran et al., 2019). Additionally, the diversity of the gut microbiota community was reported to be significantly reduced in AD patients compared with non-AD patients (Vogt et al., 2017). An increased prevalence of *Bacteroides* was proved to be independently associated with the presence of mild cognitive impairment (MCI) in patients without dementia based on graphical modeling and multivariable logistic regression analysis in a cross-sectional study (Saji et al., 2019). Besides, the latest research has shown a sex-dependent association between gut microbiota and cognitive impairment in a mice model of AD, indicating that sex itself may exert a specific impact on the composition of the microbiota in AD pathology (Cuervo-Zanatta et al., 2021).

Gut microbiota may participate in the development and progression of AD in multiple ways. A key player in the complex pathogenesis of AD in neuroinflammation, which is characterized by the excessive activation of microglia, leading to significant changes in their structure and function and resulting in the production of large amounts of pro-inflammatory factors in the CNS. Emerging evidence from both animal and human studies supports an association between dysbiosis and microglia activation during AD development (Vogt et al., 2017; Zhuang et al., 2018). Induced by certain gut bacteria, Aβ protein deposited in the brain can bind to the CD14 receptor, methyl peptide receptors, and Toll-like receptors 4 (TLR4) on the surface of microglia. This activates these cells, which release large amounts of pro-inflammatory factors such as IL-6 and TNF-α, thereby activating astrocytes, and further aggravating inflammatory responses in the brain (Lehnardt, 2010). Gut dysbiosis can also alter the production of tight junction proteins in the intestinal mucosa, which can destroy the intestinal barrier, leading to LPS leakage into the blood circulation and causing neuroinflammation (Cani et al., 2008). Recent evidence has implicated infiltrating peripheral immune cells, such as CD4^+^ and CD8^+^T cells in AD-associated neuroinflammation (Merlini et al., 2018). Wang X. et al. (2019), suggested that the combination of specific bacteria (e.g., Th1/M1-associated bacteria), amino acids (e.g., phenylalanine and isoleucine), and the composition of brain-infiltrating immune cell (e.g., Th1- dominant) may serve as an early diagnostic biomarker for MCI in AD patients.

Amyloid-β accumulates in the cerebral cortex and hippocampus of AD patients to form toxic senile plaques, which play an important role in the pathogenesis and progression of this disease. A variety of bacterial species resident in the human gut can produce amyloid, including *Pseudomonas*, *Streptomyces*, *Bacillus*, and *Escherichia coli*, which may contribute to both the systemic and CNS-associated amyloid burden in aging humans (Zhao and Lukiw, 2015). The amyloid secreted by *E.coli* has a similar structure and immunogenicity to that of Aβ42, and can promote the release of pro-inflammatory factors by binding to the Toll-like receptors 2 (TLR2) on the surface of microglia and subsequently exacerbate the inflammatory response in the AD brain (Hill and Lukiw, 2015). Tau protein hyperphosphorylation is another pathological feature of AD. Gut microbiota may directly or indirectly regulate tau hyperphosphorylation through multiple mechanisms: (1) The interaction between deposited Aβ and tau protein could induce tau hyperphosphorylation, and microbes can influence this process by regulating Aβ deposition. (2) Through alleviating oxidative stress-induced damage by producing antioxidants or enhancing the activity of superoxide dismutase (SOD) and glutathione (GSH), gut microbiota can to some extent inhibit tau hyperphosphorylation (Wall et al., 2014; Yan et al., 2016). (3) Gut microbiota can interfere with the insulin signaling pathway by inducing inflammation and promoting adipogenesis, leading to the activation of glycogen synthase kinase 3β (GSK-3β), which promotes tau hyperphosphorylation. (4) The systemic inflammation induced by LPS secreted by specific bacteria can accelerate the hyperphosphorylation of tau protein (Savignac et al., 2016).

The gut microbiota is important for the synthesis of brain neurotransmitters (such as GABA and 5-HT), BDNF, and SCFAs, which are all associated with AD (Hu et al., 2016). GABA is a major inhibitory neurotransmitter in the human CNS. Studies have shown that the GABA level in the gut is affected when the gut microbiota is disturbed, especially when the numbers of *Bifidobacteria* and *Lactobacilli* decrease, which in turn leads to a decline in GABA levels in the CNS (Bhattacharjee and Lukiw, 2013). Glutamate is a crucial excitatory neurotransmitter in the human CNS, and is known to bind the N-methyl-D-aspartate (NMDA) receptor, an important regulator of neuronal activation, dendritic and axon structure, and synaptic plasticity. Neufeld et al. (2011) reported that the mRNA expression of NMDA receptor subunit 2B (NR2B) was significantly downregulated in the hippocampus of sterile mice, indicating that a correlation exists between the gut microbiota and the expression of the NMDA receptor. SCFAs are important metabolic products of gut microbiota, regulating the metabolism of free fatty acids, glucose, and cholesterol in the body through various cell-signaling cascades involving G protein-coupled receptors (Den Besten et al., 2013). SCFAs also serve as important modulators in immunity via NF-κB signaling, regulating the responsiveness of microglial cells to LPS (Jiang et al., 2017). Besides, SCFAs exert neuro-modulatory and epigenetic effects through histone acetylation and were shown to improve cognitive function in animal models of neurodevelopmental and neurodegenerative diseases (Stilling et al., 2014). Given these important physiological functions of SCFAs, reduced SCFA production by the gut microbiota of AD patients may represent another mechanism underlying the development of AD (Zhuang et al., 2018).

### Parkinson’s Disease

Parkinson’s disease is a long-term degenerative disorder of the CNS, and is characterized by the abnormal aggregation of α-synuclein and the degeneration and necrosis of substantia nigra dopaminergic neurons. The clinical manifestations of PD include both motor symptoms and autonomic non-motor symptoms such as cognitive impairment and sleep disturbance. Several lines of evidence indicate that the gut may be involved in the origin of PD. For instance, gut symptoms appear in the early stages of PD, while α-synuclein misfolding and aggregation also initially occur in the ENS and dorsal motor nucleus of the vagus nerve (Braak and Del Tredici, 2017). Furthermore, LPS released by gut bacteria can also modulate α-synuclein aggregation, which is a well-characterized interaction associated with this alternative pathway of PD progression (Bhattacharyya et al., 2019). Holmqvist et al. (2014) for the first time demonstrated that various forms of α-synuclein can spread into the brain along the vagus nerve after being injected into the gut of mice, regardless of whether the injected material was a human PD brain lysate containing abnormally folded α-synuclein or recombinant forms (monomers, oligomers, or fibrils) of α-synuclein protein.

The gut microbiota is disordered in PD patients (Scheperjans et al., 2015). The abundance of *Prevotellaceae* in the gut of PD patients was reported to be 77.6% lower than that of healthy controls, and this decline may result in reduced mucin synthesis and a subsequent increase in gut permeability (Caputi and Giron, 2018; Gerhardt and Mohajeri, 2018). One study revealed that the abundance of “anti-inflammatory” butyrate-producing bacteria from the genera *Blautia*, *Coprococcus*, and *Roseburia* was significantly lower in the colonic mucosa and fecal samples of PD patients than in those of control subjects (Keshavarzian et al., 2015). *Verrucomicrobia*, *Mucispirillum*, *Porphyromonas*, *Lactobacillus*, and *Parabacteroides* were reported to be the most abundant taxa in PD patients, while *Prevotella* is more abundant in the gut of healthy individuals. The abundance of *Bacteroides* is significantly higher in PD patients without tremor symptoms than in those presenting with tremors, and increases with motor symptom severity (Lin et al., 2019). This study indicated that a correlation existed between changes in the gut microbiota of PD patients and the severity of their clinical phenotype. Epidemiological studies involving Danish and Swedish patients have also shown that truncal vagotomy is protective against PD (Svensson et al., 2015; Liu et al., 2017), providing clinical evidence for a link between gut microbiota and this disease. Additionally, a preclinical study using a mouse model of PD showed the beneficial effect of a nutritional supplement containing prebiotic fibers (fructo-oligosaccharide [FOS] and galacto-oligosaccharide [GOS]), on motor, cognitive, and gut symptoms (Perez-Pardo et al., 2017). Gut bacteria may be implicated in PD through several pathways. Gut dysbiosis can induce intestinal inflammation, leading to the accumulation of α-synuclein in the ENS, which may subsequently spread to the CNS through the vagus nerve (Houser and Tansey, 2017). Additionally, SCFAs were shown to be significantly reduced in the gut of PD patients, which may result in mucosal barrier impairment and increased gut permeability, thereby affecting the immune system, ENS, and CNS, and exerting a profound effect on the condition of PD patients (Vizcarra et al., 2015). Additionally, a recent study identified significant changes in the phage/lactic acid bacteria ratio in PD patients. The bacteria are known to produce dopamine and regulate intestinal permeability and are major factors implicated in PD pathogenesis (Tetz et al., 2018). However, more research is needed to understand the mechanism underlying how the gut microbiota affects the development of PD.

### Hypertension

Hypertension (HTN) is a major risk factor for cardiovascular, cerebrovascular, and kidney diseases in the elderly. Longitudinal studies have indicated that HTN during midlife may be a risk factor for subsequent cognitive decline and dementia (Wysocki et al., 2012; Walker et al., 2019). HTN may be induced by a complex interplay between genetic and environmental factors; however, the precise cause of this morbidity has not been elucidated to date (Li et al., 2017). Genetic, environmental, and dietary factors profoundly influence both gut microbiota and blood pressure, suggesting a link between gut dysbiosis and HTN (Yang et al., 2015). Li et al. (2017) found dramatically decreased microbial richness and diversity, a *Prevotella*-dominated gut enterotype, distinct metagenomic composition with reduced numbers of beneficial bacteria, and disease-linked microbial function in both pre-hypertensive and hypertensive human populations compared with that of healthy controls. Additionally, blood pressure is elevated in GF mice after the transplantation of fecal microbiota from hypertensive humans, demonstrating that gut microbiota can directly influence the blood pressure of their host. Diet is an important factor in the onset of HTN. A recent study reported that the detrimental effects resulting from low-fiber Westernized diets may underlie hypertension, which may be due to a deficiency in SCFA production by gut microbiota (Kaye et al., 2020). Evidence has suggested that LPS overproduction by gut bacteria may be directly linked to HTN development, whereas amino acid biosynthesis, fatty acid utilization, and purine metabolism by bacteria might have a role in HTN prevention (Li et al., 2017). Overall, despite the mechanism being undefined, gut dysbiosis has been closely linked with hypertension, which is likely to be associated with bacterial metabolites, such as SCFAs.

### Atherosclerosis

Atherosclerosis is the most common vascular brain pathology in the elderly, and is characterized by the formation of atherosclerotic plaques mainly consisting of accumulated modified lipids, hyperplastic smooth muscle cells and collagen fibers (Gui et al., 2012). Studies have shown that cerebral atherosclerosis can increase the risk of dementia, particularly that associated with AD (Arvanitakis et al., 2016). Accumulating evidence suggested that a link exists between gut microbiota and atherosclerosis development, and changes in the function and composition of bacterial population may increase the risk for atherosclerosis through complex mechanisms (Drosos et al., 2015). Compared with that of healthy controls, the microbiota of atherosclerosis patients exhibits an increased abundance of *Enterobacteriaceae* and *Streptococcus spp*., resulting in a less fermentative and more inflammatory gut environment (Jie et al., 2017). Gut microbiota might be involved in the progression of atherosclerosis primarily through modulating inflammation and the production of microbial metabolites such as trimethylamine-N-oxide (TMAO), SCFAs, and bile acids (Brown and Hazen, 2018). TMAO, a gut microbe-dependent metabolite, was shown to be important for the development of atherosclerosis through the regulation of the host’s immune system (Jandhyala et al., 2015) and cholesterol metabolism (Geng et al., 2018), contributing to oxidative stress (Mohammadi et al., 2018) and inflammation (Ma et al., 2017), and increasing the risk of thrombosis (Zhu et al., 2016). Studies have found that the level of TMAO in plasma can be reduced by remodeling the gut microbiota through the intake of probiotics and prebiotics (Qiu et al., 2018; Tenore et al., 2019).

### Type 2 Diabetes Mellitus

Diabetes mellitus (DM) is one of the most important public health challenges of the 21st century and poses a serious threat to the health of the elderly. Epidemiological studies have shown that diabetic patients are more susceptible to dementia than healthy individuals (Sun et al., 2020). Type 2 diabetes mellitus (T2DM) is associated with an increased risk of cognitive impairment through multiple-mechanisms, among which vascular disease may be a key factor (Biessels et al., 2014). Altered gut microbial composition has been implicated in diabetes. Diabetic patients were reported to have a low abundance of butyrate-producing bacteria and reduced proportions of *Verrucomicrobiae* compared with healthy controls (Tai et al., 2015). Additionally, the abundance of *Lactobacillus* was reported to be significantly increased in T2DM patients, whereas that of *Bifidobacteria* was decreased (Sedighi et al., 2017), Another study found a markedly lower abundance of *Lactobacillus acidophilus* in fecal samples of diabetic patients, suggestive of a correlation between Lactobacillus and T2DM (Halawa et al., 2019). In addition, the amounts of *Faecalibacterium prausnitzii* and *Akkermansia muciniphila* were lower in the guts of T2DM patients than in those of non-diabetic patients (Fassatoui et al., 2019). The effect of microbiota on T2DM has been proposed to be mediated through mechanisms involving modifications in butyrate and incretin secretion (Baothman et al., 2016). Although there is evidence for a link between gut dysbiosis with T2DM, differences in research populations, sequencing technologies, analysis methods, diets, and drugs utilized have led to varied results. Consequently, further studies are needed to uncover the exact relationship between T2DM and the gut microbiomes.

## Potential Intervention Strategies Targeting Gut Microbiota in Brain Aging and Cognitive Impairment

### Dietary Fiber

Lifestyle greatly influences diet structure in humans, while the gut microbiome can rapidly adapt to dietary alterations (David et al., 2014). Specific diets (low-fermentable, oligo-, di-, mono-saccharides, and polyols [FODMAPs] and gluten-free diet [GFD]) and eating habits can have a positive effect on balanced microbiota composition and thus contribute to the enhancement of cognitive functions, important for any learning process (Novotny et al., 2019). Numerous evidences have shown that certain types of dietary fiber can regulate the number of microorganisms and their metabolites, i.e., the intake of fructan and GOS can increase the abundance of *Bifidobacteria* and *Lactobacilli* (So et al., 2018). A high-fat diet not only leads to weight gain, but also increases gut permeability and systemic inflammation levels, and reduces defense functions (Lam et al., 2012). In contrast, dietary fiber can correct the composition of the gut microbiota and promote the production of SCFAs (Bishehsari et al., 2018). SCFAs, including butyric acid, acetic acid, and propionic acid, are beneficial for metabolism and physiological health, helping to maintain the acidic environment of the intestinal cavity, increasing the abundance of beneficial bacteria, promoting mucus secretion, and sustaining the barrier function of the intestinal mucosal. Butyric acid can cross the BBB and function as an anti-depressive ability (Han et al., 2014), and can also help prevent colon cancer by inhibiting histone deacetylase (Hamer et al., 2008). Propionic acid contributes to cholesterol synthesis, plays an important role in liver gluconeogenesis, and exerts a preventive effect against metastatic liver cancer (Comalada et al., 2006). Acetic acid, used in liver fat production and cholesterol synthesis, can regulate the blood supply of the colon and protect against liver cancer (Chambers et al., 2002). SCFAs may also modulate the hypothalamic-pituitary-adrenal axis by directly affecting the mucosal immune system, and further affect information transmission in the CNS through these mechanisms (Perry et al., 2016). De Filippo et al. (2010) compared the fecal microbiota of African children who consumed a high fiber diet and European children who consumed a Westernized diet, and found that the African children had greater gut microbial diversity and abundance of SCFA-producing bacteria and reduced quantities of potentially pathogenic strains compared with their European counterparts. This suggested that diet plays a dominant role in shaping the gut microbiota and highlighted the beneficial effect of dietary fiber on maintaining a healthy intestinal tract.

### Probiotics and Prebiotics

Using probiotics and prebiotics to regulate the gut microbiota represents a viable strategy to delay aging. In addition to providing health benefits for individuals with underlying pathologies, probiotic supplementation can also improve the gut microbiota composition in healthy adults (Khalesi et al., 2019). The probiotics currently used in humans mainly include *Lactobacillus*, *Bifidobacterium*, *S. thermophilus*, *Enterococcus*, and *Bacillus*. Probiotics can regulate the release of neurotransmitters, increase the levels of tryptophan-derived neurotrophic factors, and contribute to the prevention and early treatment of cognitive dysfunction-related diseases (Bhattacharjee and Lukiw, 2013). Certain *Lactobacillus* and *Bifidobacterium strains* secrete important neurotransmitters such as GABA, acetylcholine, dopamine, or 5-HT, which play important roles in controlling the neural excitatory-inhibitory balance, mood, cognitive functions, and learning and memory processes (Bravo et al., 2011; Barrett et al., 2012; O’Mahony et al., 2015). In animal models, *Lactobacillus helveticus* NS8 can increase the expression of 5-HT and BDNF in the hippocampus, and induce significant improvements in cognitive function (Liang et al., 2015). Prebiotics, mainly including FOS, GOS, manno-oligosaccharides (MOS), and xylo-oligosaccharide (XOS), are non-digestible food ingredients that can regulate the gut microbiota by stimulating the growth of some beneficial bacteria (Duncan and Flint, 2013). GOS administration by gavage significantly increased the expression of BDNF in the brains of rats (Savignac et al., 2013). A recent study showed that prebiotic supplementation (FOS-inulin) can inhibit Con A-induced systemic inflammation in middle-aged mice, particularly by reducing TNF-α, indicating that prebiotics might have specific systemic effects on the immune priming of T and NK cells (Boehme et al., 2020).

Probiotics can improve intestinal defense mechanisms against pathogenic microorganisms and enhance the immune system (Rooks and Garrett, 2016). Studies have demonstrated that probiotic intake can reduce systemic inflammation by decreasing the levels of pro-inflammatory cytokines, such as IL-1β (Ait-Belgnaoui et al., 2012; Luo et al., 2014), IL-6, and TNFα (Ait-Belgnaoui et al., 2012), as well as those of microglial activation markers (Ait-Belgnaoui et al., 2014). In the CD1 mouse, prebiotic treatment can significantly suppress the LPS-induced inflammatory response, decrease the expression of IL-1β in the cortex, and increase the expression levels of 5-HT and those of components of the neuronal protective glutamatergic system (Savignac et al., 2016). In a mouse model of cognitive impairment, gastric infusion of *Lactobacillus pentosus* var. *plantarum* C29 significantly improved D-galactose-induced memory dysfunction, increased the expression of BDNF in the brain, and decreased the expression of the senescence marker p16 and that of the inflammation markers TNF-α, p-p65, p-foxo3a, cyclooxygenase-2 (COX-2), and inducible nitric oxide synthase (iNOS), indicating that C29 may ameliorate aging-related memory impairment and inflammation (Woo et al., 2014). A recent study has revealed that the SLAB51 formulation, a mixture of bifidobacteria and lactobacilli, can counteract the detrimental effect induced by 6-hydroxydopamine (6-OHDA) *in vitro* and *in vivo* models of PD through modulating the BNDF pathway, increasing the PPARγ, activating the Nrf2/HO-1 pathway and inhibiting NFκB, which suggested that SLAB51 can be a promising candidate for PD prevention or treatment or as coadjuvant therapy (Castelli et al., 2020). In addition, probiotics may delay senescence through their antioxidant and free radical scavenging abilities (Li et al., 2012). *Lactobacillus fermentum* can increase the total serum antioxidant capacity in the pig by reducing malondialdehyde levels and increasing those of SOD and glutathione peroxidase, and has the capacity for scavenging free radicals (Wang et al., 2009).

### Fecal Microbiota Transplantation

Fecal microbiota transplantation (FMT) is defined as the transplantation of the functional bacteria present in the feces of healthy people into the gastrointestinal tract of patients to rebuild the gut microbiota and treat both intestinal and extra-intestinal diseases. FMT has been used in the treatment of and exploratory research into various microbiota-related diseases, such as *Clostridium diffcile* infection (CDI), and is regarded as a medical breakthrough. A recent study reported that transferring the gut microbiota of aged mice to young GF mice promoted inflammation in the small intestine of the latter and enhanced the leakage of inflammatory bacterial components into the systemic circulation (Fransen et al., 2017). Ridaura et al. (2013) transplanted the fecal microbiota of adult twin pairs of mice, one obese and one not, into GF mice fed a low-fat chow, and found that mice transplanted with the fecal microorganisms of obese mice became obese, while those transplanted with the fecal microorganisms of lean mice became lean, revealing that microbiota transplantation can change the composition of the host microbiota. Another study demonstrated that gut microbiota may play a key role in modulating the life span of vertebrates. Recolonizing the gut of middle-aged African turquoise killifish with bacteria from young killifish increased life span and delayed behavioral decline, while also helping to maintain an overall healthier physiological status, and a highly diverse and young-like gut microbial community (Smith et al., 2017). In summary, the transplantation of gut microbiota has been shown to have the potential to prolong life; however, the unraveling of the underlying mechanisms requires extensive further investigation.

## Conclusion and Future Perspectives

The association between gut microbiome and conditions of the host are complicated. The composition of gut microbiota and cellular metabolism varies with aging. Whether the alteration of gut microbiota is inducer of or consequence of cognitive disorder and its mechanisms need to explored. Emerging evidences have emphasized the importance of the preservation of a healthy microbiome to maintain brain functions during aging. They have supported a causal or contributory role of gut microbiota in the progress of cognitive impairment. The contribution of gut microbiota in the origin of PD provided clear confirmation of causality, evidenced by early appearance of α-synuclein inclusions in the ENS, glossopharyngeal and vagal nerves, and the reduced risk for PD in vagotomized individuals (Braak et al., 2003; Shannon et al., 2012; Svensson et al., 2015). Moreover, it was demonstrated that a 12-week consumption for probiotic (*Lactobacillus acidophilus*, *Lactobacillus casei*, *Bifidobacterium bifidum*, and *Lactobacillus fermentum*) can positively affect cognitive function and some metabolic statuses in the AD patients through a randomized, double-blind, and controlled clinical trial (Akbari et al., 2016). GF mice and antibiotic-induced gut dysbiosis are two methods to investigate the causality in gut microbiota-brain relationships (Frohlich et al., 2016). GF mice showed changes in anxiety-like, social and cognitive behavior compared with SPF mice (Diaz Heijtz et al., 2011; Desbonnet et al., 2014; Stilling et al., 2014). The GF APP transgenic mice showed a drastic reduction of cerebral Aβ amyloid pathology compared with control mice, while the Aβ pathology increased after colonization with microbiota from conventionally raised APP transgenic mice (Harach et al., 2017). One study has showed the pseudo GF mice that received fecal bacteria transplants from senescence-accelerated mouse resistant 1 (SAMR1) mice but not from senescence accelerated mouse prone 8 (SAMP8) mice (a mouse model of AD) showed improvements in behavior and in α-diversity and β-diversity indices of microbiota (Zhan et al., 2018). Furthermore, the microbiota dysbiosis induced by antibiotics in mouse model was verified to be associated with the dysregulation of cerebral signaling molecules, including reduced BDNF, increased neuropeptide Y and serotonin transporter, which subsequently led to cognitive impairment (Frohlich et al., 2016).

However, changes of gut bacterial composition may also simply be a consequence of the disease state. Several systems are at work to ensure the efficient functioning of the gut. The CNS, ENS, the sympathetic and parasympathetic branches of the autonomic nervous system, and neuroendocrine and neuroimmune pathways are all involved in communication with the gut microbes (Cryan and Dinan, 2012). In the BGM axis, the brain can directly or indirectly affect the composition and function of the gut microbiota by releasing signal molecules through lamina propria cells or modulating motility, secretions, and permeability of the gastrointestinal tract (Cryan et al., 2019).Therefore, it is also reasonable to assume that the neuronal dysfunction, the major phenotype of neurodegenerative diseases, may contribute to the microbiota dysbiosis. A latest study supported that gut microbiota-dependent metabolite TMAO or its predecessors including choline, carnitine, and betaine do not play causal roles in the development of AD through a bidirectional mendelian randomization approach (Zhuang et al., 2021). From another point of view, both these disease models that mentioned above have limitations. As a highly artificial model, GF mice also display alterations in the blood-brain barrier and brain ultrastructure (Diaz Heijtz et al., 2011; Braniste et al., 2014) and their physiologic deficits caused by the life-long absence of microbiota are likely to be dampened by compensatory processes (Frohlich et al., 2016). As in antibiotic-induced gut dysbiosis, the antibiotics are likely to evoke systemic effects, or even act directly on the brain rather than from gut to brain. All these factors may shield the real cause behind the outcomes in probing the microbiota-dependent effects.

Although the mouse models possess plenty of advantages in the research of neurodegenerative disorders and many therapeutic approaches show considerable efficacy, there is still no effective treatment of these diseases. This may, at least in part, be related to the fact that these models are highly artificial and only mimic selected aspects representing the human disease. Pet species may be a rational model to study cognitive disorders because (i) disease phenotypes develop spontaneously as they are usually maintained by their owners, (ii) they are not required to be genetically manipulated, and (iii) the long life span and late onset of the conditions is a great parallel to human patients who are also not genetically modified. In addition, the non-human primate is another promising model in investigating human aging and cognitive impairment due to the high similarity to the human brain and nervous system regardless of their high cost and longer research period (Teng et al., 2000).

On all accounts, insights into the mechanisms involved in how gut microbes affect aging and aging-related cognitive impairment may provide new directions for the diagnosis, treatment, and prevention of aging-related diseases. Gut microbiota can be a promising intervention target for aging and cognitive disorders. The future research based on microbiota-directed interventions require more well-designed, large, randomized, double-blind, and placebo-controlled clinical trials. Moreover, research needs to illustrate the physiological and pathological function of the microbiota that are present and investigate precise mechanisms by which changed microbe composition contributes to the pathophysiology of disease and by which potential probiotic bacteria exert beneficial effects on host health.

## Author Contributions

JN and HQ contributed to organizing this article and revising its content. HL was responsible for writing the manuscript and figure design. All authors read and approved the final manuscript.

## Conflict of Interest

The authors declare that the research was conducted in the absence of any commercial or financial relationships that could be construed as a potential conflict of interest.

## Abbreviations

BGM: brain-gut-microbiota
SCFAs: short chain fatty acids
PERMANOVA: permutational multivariate analysis of variance
SPD: Sample Progression Discovery
GF: germ-free
IL-10: interleukin-10
TGF-β: transforming growth factor beta
Tregs: TGF β-producing regulatory T cells
Th17: T helper 17
ILC2: type-2 lymphoid innate cells
IgA: immunoglobulin A
BMI: body mass index
CNS: central nervous system
ENS: enteric nervous system
GABA: γ-aminobutyric acid
GAD: glutamic acid decarboxylase
BBB: blood–brain barrier
GPR: G protein-coupled receptor
5-HT: 5-hydroxytryptamine
ECCs: enterochromaffin cells
SPF: specific-pathogen-free
MAMPs: microbial-associated molecular patterns
LPS: lipopolysaccharide
BLP: bacterial lipoprotein
CpG: cytosine-phosphate-guanosine
IL-1β: interleukin-1- β
IL-6: interleukin-6
TNFα: tumor necrosis factor- α
PAMPs: pathogen-associated molecular patterns
CSF: cerebrospinal fluid
BDNF: brain-derived neurotrophic factor
NGF: nerve growth factor
AD: Alzheimer’s disease
IBS: irritable bowel syndrome
PD: Parkinson’s disease
Aβ: amyloid- β
NFTs: neurofibrillary tangles
MCI: mild cognitive impairment
TLR4: Toll-like receptors 4
TLR2: Toll-like receptors 2
SOD: superoxide dismutase
GSH: glutathione
GSK-3β: glycogen synthase kinase 3 β
NMDA: N-methyl -D-aspartate glutamate receptor
NR2B: NMDA receptor subunit 2B
FOS: fructo-oligosaccharide
GOS: galactooligosaccharide
HTN: Hypertension
TMAO: trimethylamine-N-oxide
DM: Diabetes mellitus
T2DM: Type 2 diabetes mellitus
FODMAPs: low-fermentable, oligo-, di-, mono-saccharides and polyols
GFD: gluten-free diet
MOS: manno-oligosaccharides
XOS: xylo-oligosaccharide
COX-2: cyclooxygenase-2
iNOS: inducible Nitric Oxide Synthase
6-OHDA: 6-hydroxydopamine
FMT: fecal bacteria transplantation
CDI: *Clostridium difficile* infection
SAMR1: senescence-accelerated mouse resistant 1
SAMP8: senescence accelerated mouse prone 8.
